# Phytochemical Profiling, Anti-Inflammatory, Anti-Oxidant and In-Silico Approach of *Cornus macrophylla* Bioss (Bark)

**DOI:** 10.3390/molecules27134081

**Published:** 2022-06-24

**Authors:** Ali Khan, Aini Pervaiz, Bushra Ansari, Riaz Ullah, Syed Muhammad Mukarram Shah, Haroon Khan, Muhammad Saeed Jan, Fida Hussain, Mohammad Ijaz Khan, Ghadeer M. Albadrani, Ahmed E. Altyar, Mohamed M. Abdel-Daim

**Affiliations:** 1 Department of Pharmacy, University of Swabi, Ambar 94640, Pakistan; tpihsmardan@gmail.com (A.K.); aini_khan91@yahoo.com (A.P.); saeedjanpharmacist@gmail.com (M.S.J.); fida2k16@gmail.com (F.H.); ijazkhattak@uoswabi.edu.pk (M.I.K.); 2 Departement of Pharmacy, Abdul Wali Khan University, Mardan 23200, Pakistan; saeedjanpharmacist@gmal.com (B.A.); haroonkhan@awkum.edu.pk (H.K.); 3 Department of Pharmacognosy, College of Pharmacy, King Saud University, Riyadh 11451, Saudi Arabia; rullah@ksu.edu.sa; 4 Department of Biology, College of Science, Princess Nourah bint Abdulrahman University, Riyadh 11671, Saudi Arabia; gmalbadrani@pnu.edu.sa; 5 Department Pharmacy Practice, Faculty of Pharmacy, King Abdulaziz University, Jeddah 21589, Saudi Arabia; aealtyar@kau.edu.sa; 6 Pharmacy Program, Department of Pharmaceutical Sciences, Batterjee Medical College, Jeddah 21442, Saudi Arabia; abdeldaim.m@vet.suez.edu.eg; 7 Pharmacology Department, Faculty of Veterinary Medicine, Suez Canal University, Ismailia 41522, Egypt

**Keywords:** GC-MS, anti-inflammatory, antioxidant, *Cornus macrophylla*

## Abstract

The objective of the current study was to evaluate the phytochemical and pharmacological potential of the *Cornus macrophylla*. *C. macrophylla* belongs to the family Cornaceae. It is locally known as khadang and is used for the treatment of different diseases such as analgesic, tonic, diuretic, malaria, inflammation, allergy, infections, cancer, diabetes, and lipid peroxidative. The crude extract and different fractions of *C. macrophyll* were evaluated by gas chromatography and mass spectroscopy (GC-MS), which identified the most potent bioactive phytochemicals. The antioxidant ability of *C. macrophylla* was studied by 2,2′-azino-bis-3-ethylbenzthiazoline-6-sulfonic acid (ABTS) and 1,1 diphenyl-2-picryl-hydrazyl (DPPH) methods. The crude and subsequent fractions of the *C. macrophylla* were also tested against anti-inflammatory enzymes using COX-2 (Cyclooxygenase-2) and 5-LOX (5-lipoxygenase) assays. The molecular docking was carried out using molecular operating environment (MOE) software. The GC-MS study of *C. macrophylla* confirmed forty-eight compounds in ethyl acetate (Et.AC) fraction and revealed that the Et.AC fraction was the most active fraction. The antioxidant ability of the Et.AC fraction showed an IC_50_ values of 09.54 μg/mL and 7.8 μg/mL against ABTS and DPPH assay respectively. Among all the fractions of *C. macrophylla*, Et.AC showed excellent activity against COX-2 and 5-LOX enzyme. The observed IC_50_ values were 93.35 μg/mL against COX-2 and 75.64 μg/mL for 5-LOX respectively. Molecular docking studies supported these in vitro results and confirmed the anti-inflammatory potential of *C. macrophylla. C. macrophylla* has promising potential as a source for the development of new drugs against inflammation in the future.

## 1. Introduction

Inflammation is a degenerative process causing low molecular weight catabolic entities local accumulation, resulting in tissue osmotic pressure increases attracting extra fluids, with or without production of sufficient heat for tissue temperature elevation [1]. Some of the dominant pathological conditions are associated with inflammation as tissue swelling (tumor), tissue temperature increases, inflammation site redness, and noxious stimuli creating an intense sensation, and function loss of the affected organ [2]. Inflammation mechanism on the molecular level is a complicated process, initiated by the identification of a specific molecular pattern linked with injury or infection. Several regulators mediate the complete process of the inflammatory responses which are involved in the regulation of various pro-inflammatory molecules [3].

Reactive oxygen species (ROS) can be generated in nearby tissues as a result of the host’s immunological and inflammatory responses to pathogens. These include DNA damage, lipid peroxidation [4], oxidation of key enzymes, release of pro-inflammatory cytokine which is stimulated by macrophages and monocytes, protein damage, and other processes. These all contribute to tissue damage caused by reactive oxygen species [5]. Inflammatory disorders have prompted extensive research into the mechanisms of ROS-mediated tissue destruction [6]. The hydroxyl radical, superoxide anion, nitrous oxide radical hypochlorous acid, singlet oxygen, and hydrogen peroxide are the main prominent ROS involved in inflammatory tissue damage [7]. Many of the antioxidants which are secreted locally at the site of infection and other tissues such as epithelium provide protection against such species [8].

Medicinal plants used in traditional medicine have a long history in developing countries [9]. They are generally used in many countries as folk medicine to treat different inflammatory conditions and inflammations of skin tissue [10]. The *C. macrophylla* species of the genus *Cornus* belongs to the family *Cornaceae*. Locally it is known as khadang and is used in folklore for the treatment of a variety of diseases [11]. The fruit of *C. macrophylla* is a traditionally important plant and has been used as a remedy for inflammation. The *C. macrophylla* plant is also used in various pathological conditions as an analgesic, diuretic, and as a tonic for the preservation of foods [12]. The fruit of the *C. macrophylla* plant is used for diverse ailments like allergic reactions, malarial infections, diabetes, cancer, and as a lipid peroxidative or anti-inflammatory agent [13,14]. The Genus *Cornus* encompasses 58 species of mostly small trees and hermaphroditic shrubs which are widely distributed in rarely tropical and temperate regions of the northern hemisphere with a range covering North and Central America, Asia, Australia, and Europe [15,16]. *Cornifructus* aqueous extracts mainly repressed Nitric Oxide (NO) production and Prostaglandin E_2_ (PGE_2_) synthesis due to inhibition of the lipopolysaccharide-induced expression of COX-2 and inducible NO synthase (iNOS) in murine macrophagic cells [17]. In the current investigation, we explored the *Cornus macrophylla* bioss. (Bark) for the first time for their antioxidant and anti-inflammatory potential along with GC-MS analysis.

## 2. Results and Discussion

Inflammation is a complex sequence of molecular reactions and cell functions that is intended to heal tissue after a small skin cut or repair tissue after childbirth and treat a variety of serious burns [18]. A sequence of events with dilatation of venules and arterioles increased vessel permeability and blood flow with leukocyte percolation into the tissues characterize an inflammatory reaction at the cell and molecular levels [19]. The inflammatory pathway is preconfigured and patterned and is the only method for tissue regeneration following injury [20]. The genus *Cornus* is well known for its medicinal properties. The crude extract of *Cornus kousa* inhibited COX-1 and 2 enzymes activities by 24% and 47%, 40% and 37%, 20% and 37%, 52% and 63%, and 48% and 55% respectively, at 231, 215, 226, 258, and 217 µM [21] while the crude extract of our plant, i.e., *Cornus macrophylla* showed 69.6% inhibitory activity against the COX-2 enzyme. *Cornus alternifolia*, *Cornus contraversa*, *Cornus*, and *Cornus florida* also possess anti-inflammatory activity by inhibiting COX-1 and COX-2 enzymes. This anti-inflammatory activity is due to the compounds, i.e., anthocyanins that inhibited the COX-1 enzyme by 39% and 49% while the COX-2 enzyme by 54% and 48% respectively at 100 µg/mL [22]. COX-2 protein expression was decreased by pretreatment with *Corni fructus* extract at 100 µg/mL and 1000 µg/mL (8.14 ± 1.04 and 6.09 ± 0.93 respectively). *Corni fructus* extract displayed significant inhibition of COX-2 protein expression [17]. *Cornus walteri* is another plant of this *Cornus* genus that also showed good anti-inflammatory activity, in LPS-induced assay, by inhibiting interleukin-1, IL-6, IL-10, and TNF-α. The extract concentrations used were100 mg/kg, 200 mg/mL, and 300 mg/mL settings [23]. *Cornus sanguinea* leaves extract inhibited COX-1 enzyme by 70.71 ± 1.88% and 79.38 ± 0.92% at 50 and 100 µg/mL. Its leaf extract had higher COX-1 and COX-2 inhibitory effect than that of the fruits extract. In a COX-2 inhibitory assay, at 100 µg/mL, leaves extract of *Cornus sanguinea* displayed significant inhibition as compared with celecoxib. The IC_50_ value of leaves extract was determined as 11.39 ± 2.39 µg/mL on COX-2 [24]. Our research plant showed that crude extracts have IC_50_ values of 130.02µg/mL while Ethyl acetate fractions possess IC_50_ values of 93.35 µg/mL compared with a standard drug, celecoxib, which had an IC_50_ value of 21.58 µg/mL. Our earlier studies on *Cornus* spp fruits indicated that anthocyanins were the most abundant bioactive compound in ripened fruits [22]. Therefore, it is evident that consumption of the *Cornus* species has the potential to contribute to overall health benefits.

### 2.1. Phytochemistry

**Figure 1:**
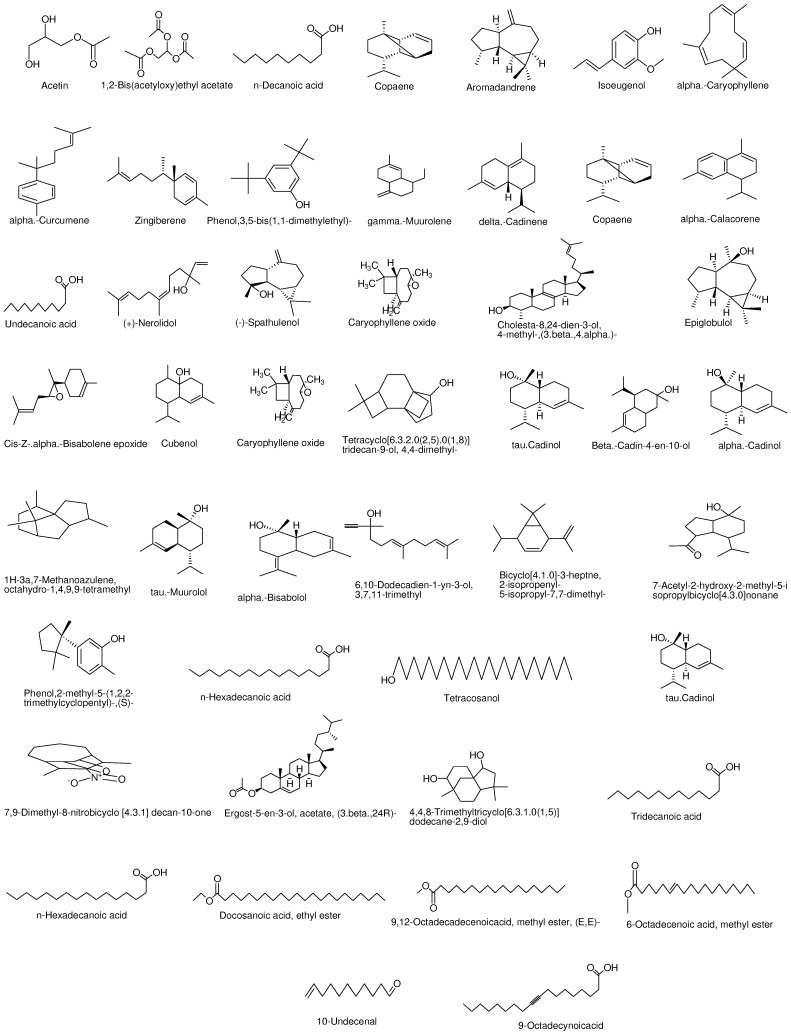
Structures of identified compound of ethyl acetate fractions.

GC-MS is the best identification technique for different constituents present in plants and various fractions. A GC-MS study of *C. macrophylla* exhibited the identification of 48 phytochemical compound (the structure of which being shown in Figure 1) ethyl acetate fractions, which could further add a contribution to the medicinal activity of plants. The different phytochemicals were confirmed by peak area, molecular structure, and retention time. These active phytochemicals along with their molecular structure, peak area, retention time, and concentration percentage are expressed in Table 1. Acetin is the first compound identified with less retention time (9.154 min) and the last phytochemical with the longest retention time value (29.310 min) was 9-Octadecynoicacid. The mass spectra of identified compounds from *C. macrophylla* are presented in Supplementary Materials Figure S1. Hexadecanoic acid is known for its anti-inflammatory and antioxidant activity [25]. Isoeugenol also possesses antioxidant activity [26]. Eugenol and delta cadinene are essential oils detected in the GC-MS of our research plant, which have marked antioxidant activity [27].

### 2.2. Antioxidant Activity

In human pathological and physiological processes, reactive oxygen species (ROS) perform a critical role [28]. Usually, there seems to be a balance between both the free radicals production and endogenous antioxidant defense mechanisms. If this discrepancy occurs, oxidative stress can occur. This level of oxidative stress can harm all critical cellular constituents such as proteins, DNA, and membrane lipids, leading to cell death [29,30,31]. Reactive oxygen species (e.g., hydroxyl radicals, hydrogen peroxide, etc.) formation is increased when the body is under huge stress. Endogenous enzymatic and non-enzymatic antioxidant molecules are unable to cope with the ROS overload, resulting in metabolic imbalances, cell damage, and health issues [30]. This can result in a variety of secondary complications including cardiovascular disease, diabetes, inflammation, degenerative diseases, cancer, anemia, and ischemia [32]. Natural compound-based antioxidants play a prophylactic role in preventing the formation of free radicals, making them one of the most effective therapeutic substances for reducing illnesses caused by oxidative stress in the body. Flavonoids and phenolic compounds, in addition to having antioxidant properties, also act as anti-inflammatory agents. Shah et al. also mentioned *C.macrophylla’s* antioxidant properties [13].

### 2.3. DPPH Inhibitory Assay

The tested crude extract of *C. macrophylla* and their different fractions showed good inhibitory potential in the DPPH assay with inhibitory percentages of 92.23, 87.45, 81.90, 76.00, and 71.90 at concentrations of 1000, 500, 250, 125, and 62.5µg/mL respectively with IC_50_ value of 17.72 µg/mL. After crude extract analysis, the n-Hexane fraction showed marked inhibitory potential in the DPPH assay with 87.63%, 82.45%, 76.53%, 71.42%, and 65.68% inhibition at concentration ranges of1000, 500, 250, 125, and 62.5µg/mL having an IC_50_ value of 20.76 µg/mL. The DCM portion exhibited marked inhibitory potential in the DPPH assay with 93.10%, 87.58%, 83.76%, 75.44%, and 68.10% inhibition at a concentration from 1000, 500, 250, 125, and 62.5µg/mL respectively with IC_50_ of 5.34 µg/mL. Ethyl acetate fraction also possesses good inhibitory potential against DPPH with 94.40%, 85.03%, 80.90%, 76.44%, and 71.22% inhibition at concentrations of 1000, 500, 250, 125, and 62.5 respectively µg/mL with IC_50_ value of 7.80 µg/mL. The aqueous fraction also possesses good inhibitory potential against DPPH with 84.37%, 80.45%, 73.37%, 67.30%, and 62.42% inhibition at concentration extents of1000, 500, 250, 125, and 62.5µg/mL respectively with IC_50_ value of 16.40 µg/mL. The reference drug used in this assay was ascorbic acid, which possesses inhibitory potential of 94.40%, 85.03%, 80.90%, 76.44%, and 71.22% inhibition at concentrationsof1000, 500, 250, 125, and 62.5µg/mL respectively with inhibitory concentration (IC_50_) value of 4.32 µg/mL Table 2.

### 2.4. ABTS Activity

The tested crude extract of *C. macrophylla* and their different fractions showed good inhibitory potential in the ABTS assay with the inhibitory percentages of 83.13, 78.83, 72.70, 66.43, and 61.06 at concentrations of 1000, 500, 250, 125, and 62.5 µg/mL respectively with a calculated IC_50_ value of 19.34 µg/mL. After crude extract analysis, the n-Hexane fraction showed marked inhibitory potential in ABTS assay with 89.37, 84.44, 77.51, 72.28, and 67.46 percent inhibition with an estimated IC_50_ value of 16.76 µg/mL. The DCM fraction showed marked inhibitory potential in the ABTS assay with 82.33, 76.33, 72.67, 70.00, and 68.60 percent inhibition showing an IC_50_ value of 4.06 µg/mL. The EA fraction also possesses good inhibitory potential against ABTS with 86.91, 81.26, 76.00, 71.54, and 68.76 percent inhibition with an IC_50_ value of 9.54 µg/mL. The aqueous portion of *C.macrophylla* also possesses good inhibitory potential against ABTS with 86.91, 81.26, 76.00, 71.54, and 67.76 percent inhibition with an IC_50_ of 12.43 µg/mL. The standard drug used in this assay was ascorbic acid, which possesses inhibitory potential of 91.90, 87.08, 82.40, 77.61, and 75.45 percent inhibition at concentrations of 1000, 500, 250, 125, and 62.5 µg/mL respectively with an IC_50_ value of 3.11µg/mL as shown in Table 2.

### 2.5. Anti-Inflammatory Activity

In our experiments, crude extract and different fractions of *C. macrophylla* bark showed excellent anti-inflammatory activity against 5-lipooxygenase and cyclooxygenase 2 enzymes, which are active mediators of inflammation.

### 2.6. 5-Lipoxygenase (5-LOX) Enzyme Inhibitory Assay

Crude extract of *C. macrophylla* and their different fractions showed good inhibitory potential against the 5-LOX enzyme. Crude extract showed inhibition of 67.44, 61.87, 55.83, 50.23, and 44.29 percent at concentrations of 1000, 500, 250, 125, and 62.5 µg/mL respectively with the IC_50_ value calculated as 122.79 µg/mL. Ethyl acetate fraction was the most potent and active fraction with the significant inhibitory potential of 71.24, 65.43, 59.48, 54.47, and 47.47 percent inhibition with an IC_50_ value of 75.64 µg/mL. The n-Hexane fraction showed 71.33, 63.03, 49.00, 42.67, and 33.00 percent inhibition at same concentration with a measured IC_50_value of 218.83 µg/mL. The estimated inhibitory potential of aqueous fraction against the LOX enzyme with 67.73, 57.42, 47.39, 41.36, and 29.15 percent with IC_50_ values of 277.91 µg/mL. The DCM fraction also acquired good inhibitory potential against the 5-LOX enzyme with 77.00, 69.26, 65.89, 58.36, and 51.47 percent inhibition with an IC_50_ value of 49.52 µg/mL. The positive control used in this protocol was Montelukast, which has marked inhibition against the LOX enzyme with 83.53, 78.62, 73.42, 66.20, and 62.00 percent inhibition at concentrationsof1000, 500, 250, 125, and 62.5µg/mL with an estimated IC_50_ value of 17.30 µg/mL as in Table 3.

### 2.7. Inhibitory Assay for Cyclooxygenase (COX-2) Enzyme

The tested crude extract of *C. macrophylla* and their different fractions showed good inhibitory potential against COX-2 enzyme with 69.67, 63.20, 55.09, 49.67, and 43.40 percent inhibition at concentrations of 1000, 500, 250, 125, and 62.5 µg/mL with 93.35 µg/mL as the IC_50_ value. After analysis, the ethyl acetate extract was the most potent fraction that showed marked inhibitory potential against the COX-2 enzyme with 69.62, 63.35, 57.36, 52.62, and 46.16 percent inhibition with an IC_50_ value of 130.02 µg/mL. The n-hexane extract showed marked inhibitory potential against the COX-2 enzyme with 69.58, 61.65, 47.90, 39.03, and 31.90 percent inhibition with an IC_50_ value of 249.57 µg/mL. Aqueous also possesses good inhibitory potential against the COX-2 enzyme with 66.79, 59.67, 41.69, 35.54, and 29.00 percent inhibition with an IC_50_ value of 319.70 µg/mL. The DCM fraction also possesses good inhibitory potential against the COX-2 enzyme with 71.02, 66.69, 61.14, 56.44, and 47.72 percent inhibition with an IC_50_ value of 72.55 µg/mL. The positive control selected in this assay was celecoxib, which possesses inhibitory potential against the COX-2 enzyme of 81.85, 76.59, 69.75, 64.47, and 61.02 percent inhibition at the same concentrations with an IC_50_ value of 21.58 µg/mL Table 3.

### 2.8. Determination of TPC and TFC in C. macrophylla Bark

Using the FC method, the total phenolic content of extract was measured as 21.54 ±0.36 mg GAE/g was TPC value of *C. macrophylla*. This finding was comparable to TPC in a range of edible common plant species [33]. The TFC content of the extract was 34.16 0.54 mg QUE/g (Table 4). Furthermore, the quantity of TPC/TFC varied based on the solvent system, species/cultivars, and plant portions.

### 2.9. HPLC Chromatograms of Phenolic Acid and Flavonoid

**Figure 2:**
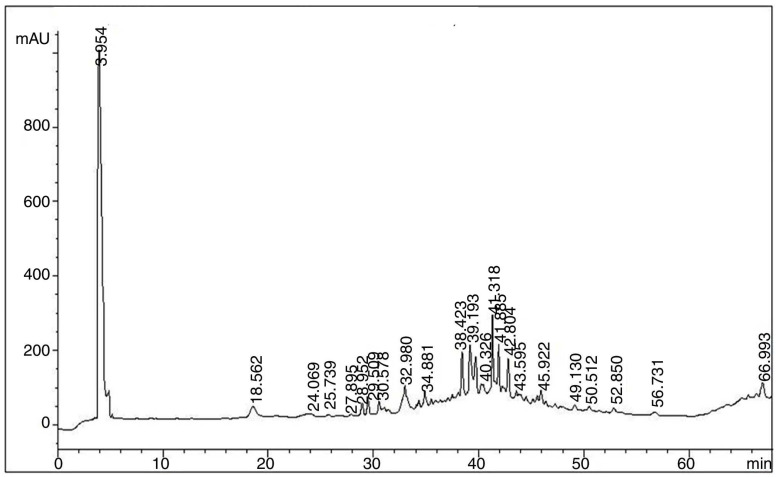
HPLC chromatogram of phenolic and flavonoids in the extract of *C. macrophylla*.

HPLC analysis allows for the simultaneous isolation and identification of a wide spectrum of phenolic acids, flavonoids, and other plant constituents [34]. We discovered that determining phenolic acids and flavonoids from *C. macrophylla* Bark using a binary solvent system in gradient mode was practicable in different methods. Caffeic acid, ferulic acid, dihydroxybenzoic acid, p-coumaric acid, chlorogenic acid, catechin, and propyl gallate obtained seven different peaks which belong to phenolic acid while rutin hydroxide, kaemferol, luteolin, narcissoside, vitexin, and myricetin obtained six peaks which belong to flavonoids (Figure 2). These peaks were analyzed and then their retention time was compared with respective standards using literature data. The amounts of these seven phenolic acids and six flavonoids were determined by developing external calibration curves and comparing them to legitimate standards.

### 2.10. Molecular Docking

**Figure 3:**
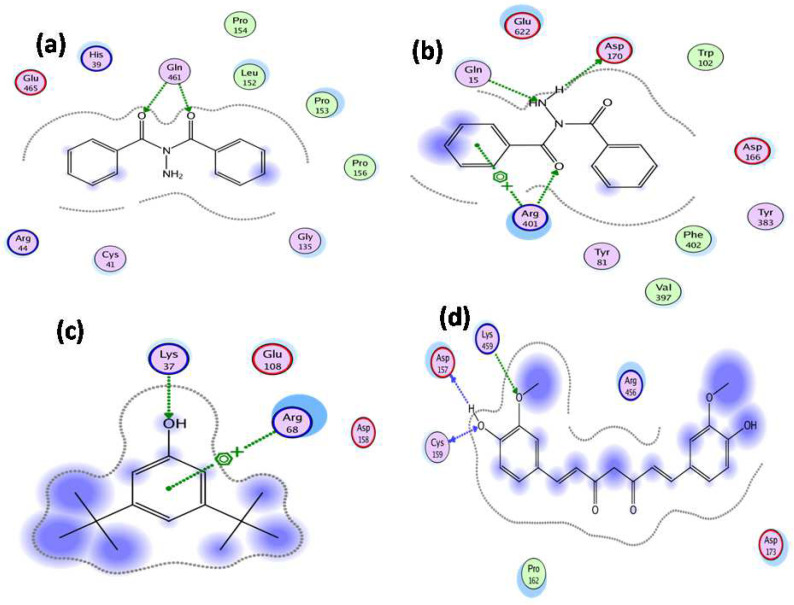
Two dimensional images (2D) of (**a**) dibezoylhydrazine with COX-2, (**b**) dibezoylhydrazine with 5-LOX, (**c**) 7-acetyl-2-hydroxy-2-methyl-5-isopropylbicyclo [4.3.0]nonane with 5-LOX, and (**d**) 6-Octadenoic acid, methyl ester with COX-2.

**Figure 4:**
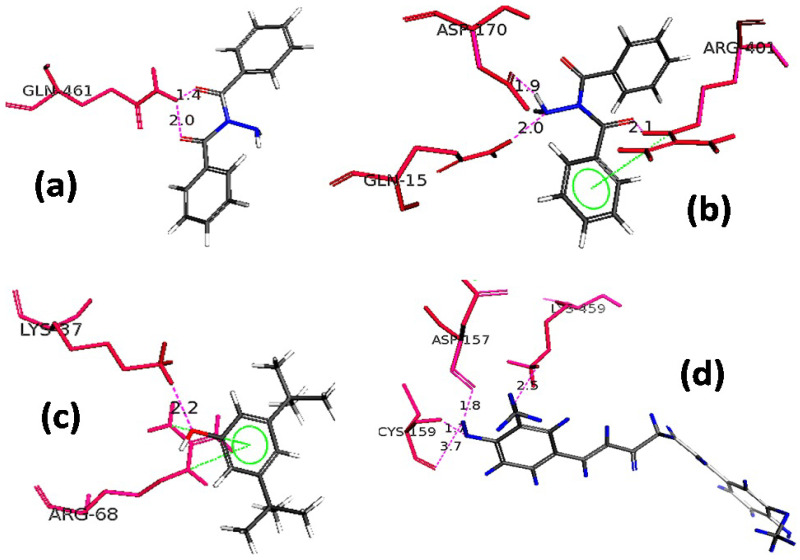
Close-up depiction of lowest energy three-dimensional (3-D) docking poses of (**a**) dibezoylhydrazine with COX-2, (**b**) dibezoylhydrazine with 5-LOX, (**c**) 7-acetyl-2-hydroxy-2-methyl-5-isopropylbicyclo [4.3.0] nonane with 5-LOX, and (**d**) 6-Octadenoic acid, methyl ester with COX-2. Bond distances in Ǻ were shown by the dotted line.

Molecular docking studies were accomplished for exploring the synergistic effect of details of active compounds of *C. macrophylla* (identified by GC-MS) on 5-LOX and COX-2 inhibitory effects. For docking simulation studies, Molecular Operating Environment (MOE, 2009) and Pymol software were used. COX-2 3D structure was obtained from protein data bank (PDB ID = 5f1a) and 5-LOX (3v92). The two-dimensional poses are shown in Figure 3 and the three-dimensional poses of identified compounds are shown in Figure 4 while the binding energy details are given in Table 5.

In this study, the identified compounds were docked into the binding site of the 5f1a and 3v92.To analyze the effects, the binding energies of the compounds were computed. These binding energy values were in the range of −7.9 to −4.1 kcal/mol for COX-2 while for 5-LOX the binding energies ranged from −8.2 to −3.8 kcal/mol. Most of the docked compounds showed binding interactions with the amino acid residues in the selective pocket of 5-LOX and COX-2. The three-dimensional structure of dibezoylhydrazine showed that oxygen molecules of the ligand caused hydrogen binding with Gln 461 residue of COX-2, having bond lengths of 2.0 Ǻ and 1.4 Ǻ. 6-Octadenoic acid, methyl ester showed that oxygen atoms of the compound possess hydrogen bonding with Lys 459 having a bond length of 2.5 Ǻ; additionally, Asp 157 and Cys 159 also showed interaction with COX-2 with bond lengths of 1.8 Ǻ and 3.7 Ǻ respectively. Dibezylhydrazine, when docked with 5-LOX, showed that two side-terminal hydrogen atoms possess hydrogen binding with Asp170 and Gln15 residues of 5-LOX having bond lengths of 1.9 Ǻ and 2.0 Ǻ respectively, while Arg401 caused a hydrogen bond with the oxygen atoms of compound (Dibezylhydrazine) having a bond length of 2.1 Ǻ and arene-cation interaction with the main nucleus. 7-acetyl-2-hydroxy-2-methyl-5-isopropylbicyclo [4.3.0] nonane, when docked with 5-LOX, showed arene-cation linkages with Arg68 residue of the enzyme while having hydrogen bonding of bond length of 2.2 Ǻ with Lys37 of 5-LOX.

## 3. Materials and Methods

### 3.1. Plant Materials

*C. macrophylla* bark was collected from the valley of Kot Manzari Baba District Malakand, Khyber Pakhtunkhwa Pakistan in March. *C. macrophylla* was identified by Professor Dr. Nisar Ahmad, Botany Department, University of Malakand and given voucher no. H. B. UOM−102 preserved in the herbarium.

### 3.2. Extraction and Fractionation

Collected bark was shaded and air-dried for 1 month, weighing approximately 4.5 Kg. The plant materials were ground into powder and soaked in solvent methanol at room temperature for 15 days with continuous stirring. After 15 days, these crude methanolic solid residues were strained by a fine Muslin cloth followed by filter paper. The solution of methanolic extract was dried by rotary evaporator at 40 °C. The final gummy extract was 510 gm. The percentage yield was 11.33% and was determined by the standard formula. The 260 gm of methanolic crude extract was preserved for pharmacological assays and the remaining extract was fractioned with dichloromethane, ethyl acetate, and n−hexane solvents based on polarities. 3 × 500 mL of water was added to 250 gm of crude methanolic extract followed by the addition of 3 × 500 mL of n−hexane to prepare solution in a separating funnel and agitated well. This mixture was suspended for a few minutes to separate the two immiscible layers of different solvents. The upper layer was separated and concentrated by a rotary evaporator and the n−hexane fraction was obtained. The process was repeated three times for the efficient removal of n−hexane fraction. For dichloromethane (DCM), fraction dichloromethane solvent was added to an aqueous solution of crude methanolic extract layer. This mixture was agitated continuously and vigorously and then suspended for some minutes until two clear immiscible layers appeared. The bottom layer of this mixture obtained was the dichloromethane layer and concentrated on a rotary evaporator for obtaining the DCM fraction. The process was repeated three times for the efficient removal of dichloromethane fraction. Again, the aqueous layer was mixed with ethyl acetate solvent and vigorously agitated for some time in the separating funnel. The mixture was suspended for some time and two clear immiscible layers were obtained. Ethyl acetate fractions were separated and concentrated by a rotary evaporator for obtaining crude ethyl acetate fraction. The process was repeated three times for obtaining an efficient amount of fraction. The final fraction obtained was considered an aqueous fraction [35,36].

### 3.3. DPPH Assay

The antioxidant effect of *C. macrophylla* was measured by utilizing DPPH (1,1−diphenyl, 2−picrylhydrazyl) free radicals [37]. Plant extract (0.1mL) in several dilutions such as62.5, 125, 250, 500, and 1000μg/mL were combined with 0.004% DPPH solution. At 517 nm, absorbance was measured after 30 min of the above addition by using a UV spectrophotometer. In this method, ascorbic acid was used as a positive standard. The scavenging percentage was determined by the following equation;

% inhibition = [absorbance of control − absorbance of the sample/absorbance of control] × 100

This activity was achieved in triplicate and GraphPad prism software (GraphPAD, San Diego, California, USA) was employed to construct inhibition curves and IC_50_(median inhibitory concentrations) was deliberate.

### 3.4. ABTS Assay

2,2−azinobis [3−ethylbenzthiazoline]-6-sulfonic acid (ABTS) protocol was applied to assess antioxidant ability of *C.macrophylla* crude extract and its different fractions. The antioxidant effect of any compound is based on the ability of that compound to scavenge ABTS free radicals and showed a mark reduction in the absorbance measured at 734 nm. The mixture of (ABTS) 7 mM and (K_2_S_2_O_4_) 2.45 mM solutions was prepared. This mixture was maintained darkened for almost 12–16 h to obtain a solution of dark color having ABTS^+^cation. For activity, a Phosphate buffer of 0.01 M having pH 7.4 was used to dilute ABTS^+^solution and regulate absorbance at 734nm. The antioxidant effect of the *C. macrophylla* was estimated by preparing a solution of extract in a concentration of 300 μ Lwith an ABTS solution of 3.0 mL. Mix solution for one minute to measure the reduction in absorbance spectrophotometrically followed by mixing these solutions for the next six min. In this assay, ascorbic acid was used as a positive control standard [38]. Above protocols were repeated three times and inhibition percent was estimated by the following formula;

% scavenging effect = (Control absorbance − Sample absorbance/Control absorbance) × 100

### 3.5. In-Vitro Cyclooxygenase (COX−2) Assay

Inhibitory potential against COX−2 enzyme was evaluated by using the standard method of Jan et al., 2019 with slight modifications. COX−2 enzyme solution was prepared at the concentration of 300 U/mL. This COX−2 solution (10μL) was activated by keeping it for 5–10 min on ice. Moreover, solution of co-factor in 50μLconcentration containing 0.9 mM glutathione, 1 mM hematin, and 0.24 mM *N*,*N*,*N*,*N*-tetramethyl-p-phenylenediamine dihydrochloride (TMPD) in 0.1 Mole Tris HCl buffer with 8.0 pH was also added to this enzymatic solution. Eventually, 20μLof *C.macrophylla* extract in different concentrations ranging from 31.25 to 1000 μg/mL and enzyme solution (60 μL) was left for five minutes at 25 °C. Similarly, by adding 30 mM arachidonic acid in quantity of 20 μL, the reaction was initiated. This solution mixture was then incubated for approximately 4–5 min. Absorbance was checked at 570 nm through a UV-visible spectrophotometer after said incubation time. The inhibitory activity of the enzyme COX−2 solution was evaluated from the absorbance value per unit time. The inhibitory concentration (IC_50_) values were estimated by plotting the enzyme inhibition against the different concentrations of plant extract. Celecoxib was used in this assay as a standard positive control [39].

### 3.6. In-Vitro 5−LIPOOXYGENASE (5−Lox) Assay

Lipooxygenase activity on *C. macrophylla* bark was achieved by using the reported standard protocol. Various conc. (31.25–1000 μg/mL) of *C.macrophylla* were prepared. Afterwards, a solution of 5 LOX enzyme with 10,000U/mL conc. was prepared. Linoleic acid 80 mM was used as a substrate. Likewise, phosphate buffer (50 mM) with pH 6.3 was primed. Various concentrations of *C.macrophylla* sample were solubilized in phosphate buffer (250 μL) and 5 LOX enzyme (250 μL) was added and incubated at laboratory temperature for a time of 5 min. Furthermore, a 1000μLsolution of a substrate (0.6 mM) was added and shaken continuously before checking the absorbance at 234 nm. These experiments were repeated in triplicate. Positive control was selected as Zileuton (Jan et al., 2020). Percentage inhibition was determined by the following formula:

Percentage Inhibition = Control Abs − Sample Abs/Control Abs × 100

The IC50 of these values was estimated by intrigue inhibitions versus the experimental sample concentrations from the subsequent equation.

### 3.7. Estimation of IC_50_ Values

The concentration of the plant extract restrained the substrate by 50% (IC_50_). The scavenging activity of radicals was evaluated by linear regression analysis amid the inhibitory percentages against the sample concentrations by using MS Excel.

### 3.8. Phytochemistry (GC−MS Analysis)

Tandem gas chromatography/mass spectrometry analysis of the methanolic extract, ethyl acetate extract, and dichloromethane extract of *C. macrophylla* was studied with Agilent USB−393752 gas chromatograph (Agilent Technologies, Palo Alto, CA, USA) with an HHP5MS 5% phenylmethylsiloxane tubular column (30 m × 0.25 mm × 0.25 μm film thickness; Restek, Bellefonte, PA) which was equipped with the Agilent HP−5973 mass selective detector in electron impact mode (Ionization energy: 70 eV) working under a similar experimental atmosphere as that exemplified for GC (Munir et al., 2020).

Identification of the classification of the components was based on the molecular mass, molecular structure, and measured fragments. Interpretation of the GC−MS was conducted via NIST (database of National Institute Standard and Technology) having about 62,000 total patterns. The molecular weight, name, and compound structure of the tested samples were ascertained. The percentage of each component was measured via evaluating its average peak area to the total areas. The spectrum of the unidentified component was evaluated comparatively with spectrum of data store in the NIST library version (2005), software, Turbomas 5.2.

### 3.9. Total Phenolic Content (TPC) Analysis for C. macrophylla Bark

The Folin Ciocalteu (FC) test was used to estimate the total phenolic content of C macrophylla [40]. The *C.macrophylla* bark was prepared in aliquots at a concentration of 1 mg/mL. Gallic acid (1–0.05 mg/mL) was used to create a calibration curve. C. macrophylla bark (1.5 mL) was added to 0.5 mL of FC reagent (3-fold diluted with distilled water), vortexed thoroughly, and left for 5 min to stand at room temperature. The mixture was treated with sodium carbonate (1 mL, 7.5% *w*/*v*) for 60 min at room temperature. The absorbance of the combined mixture was then observed at 760 nm wavelength. TPC was measured in milligrams of gallic acid equivalents (GAE)/ g.

### 3.10. Total Flavonoid Content (TFC) Analysis for C. macrophylla Bark

TFC of *C. macrophylla* bark was determined using the aluminum chloride colorimetry technique [41]. *C. macrophylla* bark extract was prepared at various concentrations (1, 0.5, 0.1, and 0.05 mg/mL). A reference calibration curve was created utilizing varying doses of quercetin (0.05–1 mg/mL). *C. macrophylla* bark (2 mL) was combined with 500 l of 10% aluminum chloride solution and 500 L of 0.1 mM sodium nitrate solution. After 30 min at room temperature, the absorbance of the reaction mixture was measured with a UV−VIS spectrophotometer at 430 nm. The flavonoids were measured in milligrams of quercetin equivalent per gram of *C. macrophylla* bark extract (mg QCE/g).

### 3.11. HPLC Analysis for Phenolic and Flavoinds Compounds

The phenolic acids and flavonoids were quantified using several internal standards such as catechin and kaempferol. After dissolving in DMSO and ethanol, stock solutions of phenolic acids and flavonoids standards were made at 1 mg/mL. For caffeic acid, ferulic acid, p-coumaric acid, chlorogenic acid, dihydroxy benzoic acid, propyl gallate, and catechin quantification, four mass concentrations (1, 0.5, 0.25, and 0.125 mg/mL) were generated independently. They were then combined to achieve a final concentration of 1, 0.5, 0.25, or 0.125 mg/mL. Similarly, flavonoid standards such as rutin hydroxide, luteolin, kaemferol, vitexin, narcissoside, and myricetin were produced in four concentrations (1, 0.5, 0.25, and 0.125 mg/mL). The standards were then blended to achieve a final concentration of 1, 0.5, 0.25, and 0.125 mg/mL. The sample was then tested in the HPLC with 10 µL [42].

### 3.12. Molecular Docking

MOE 2009 was used for molecular studies. Chem draw office was used to design the structure of all ligands. The compounds were subsequently saved for MOE loading in the mol register. Default MOE (molecular operating environment) parameters were used for energy minimization. The crystal structure COX and LOX were derived from the protein data bank (PDB). Default MOE program parameters were used to minimize energy and 3D protonate the targeted protein structure. For interaction, the ligand protein complex was studied and its 3D images were captured using PyMol visualizing system. The 2D images were depicted from MOE [43].

### 3.13. Statistical Analysis

In this paper, the required data are presented as mean ± SEM and *n* = 3. First, two-way ANOVA was applied, which was followed by the Bonferroni test for finding a significant difference between test samples and reference drug at 95% confidence interval. Data were measured significantly different with * *p* < 0.05, ** *p* < 0.01, and *p* < 0.001. ns: Experimental sample was insignificantly different as compared to standard drug *p* > 0.05.

## 4. Conclusions

It was concluded from this study that the ethyl acetate fraction showed remarkable activity against inflammation while DCM showed the most potent activity against reactive oxygen species. GCMS confirmed the presence of 48 bioactive constituents in the ethyl acetate fraction of *C. macrophylla*. The anti-inflammatory by COX/LOX and antioxidant potential by DPPH and ABTS activity of ethyl acetate showed excellent results in comparison with the standard. Molecular docking confirmed the in vitro antioxidant and anti-inflammatory potential of *C. macrophylla*. These reports will provide scientific justification for the folklore or ethnomedicinal uses of *C. macrophylla*. From our study, we concluded the interesting pharmacological effect of *C. macrophylla* which could be further investigated on the molecular level for its mechanistic pathways.

## Figures and Tables

**Table 1:** GC-MS analysis of the ethyl acetate fraction of *C. macrophylla*.

S. No.	Name	Molecular Formula	Retention Time	Peak Area	Conc. (%)
1	n-Hexadecanoic acid	C_16_H_32_O_2_	25.46	1,187,841	17.94
2	10-Undecenal	C_11_H_20_O	28.827	54,996	8.29
3	Acetin	C_5_H_10_O_4_	9.154	38,196	6.62
4	1,2-Bis(acetyloxy)ethyl acetate	C_8_H_12_O_6_	9.237	23,706	6.4
5	alpha-Cadinol	C_15_H_26_O	19.179	290,208	4.38
6	tau-Cadinol	C_15_H_26_O	18.884	249,188	3.76
7	Tridecanoic acid, methyl ester	C_14_H_28_O_2_	24.766	241,744	3.65
8	Undecanoic acid	C_11_H_22_O_2_	16.899	214,503	3.24
9	6-Octadecenoic acid, methyl ester, (Z)-	C_19_H_36_O_2_	28.109	163,759	2.47
10	alpha-Curcumene	C_15_H_22_	15.136	158,324	2.39
11	7-Acetyl-2-hydroxy-2-methyl-5-isopropylbicyclo [4.3.0]nonane	C_15_H_24_O_2_	20.992	146,133	2.21
12	delta-Cadinene	C_15_H_24_	16.137	141,079	2.13
13	Epiglobulol	C_15_H_26_O	18.081	139,893	2.11
14	Aromadandrene	C_15_H_24_	13.726	135,498	2.05
15	tau-Cadinol	C_15_H_26_O	22.285	133,874	2.02
16	1H-3a,7-Methanoazulene, octahydro-1,4,9,9-tetramethyl-	C_15_H_26_	19.538	127,638	1.93
17	n-Hexadecanoic acid	C_16_H_32_O_2_	21.328	127,019	1.92
18	Phenol,3,5-bis(1,1-dimethylethyl)-	C_14_H_22_O	15.747	119,639	1.81
19	9,12-Octadecadecenoicacid, methyl ester, (*E*,*E*)-	C_19_H_34_O_2_	27.991	115,490	1.74
20	4,4,8-Trimethyltricyclo[6.3.1.0(1,5)]dodecane-2,9-diol	C_15_H_26_O_2_	23.823	107,229	1.62
21	Docosanoic acid, ethyl ester	C_24_H_48_O_2_	26.102	106,583	1.61
22	Tetracyclo[6.3.2.0(2,5).0(1,8)]tridecan-9-ol, 4,4-dimethyl-	C_15_H_24_O	18.789	100,854	1.52
23	9-Octadecynoicacid	C_18_H_34_O_2_	29.31	98,021	1.48
24	alpha-Calacorene	C_15_H_20_	16.622	93,806	1.42
25	alpha-Bisabolol	C_15_H_26_O	19.777	76,084	1.15
26	6,10-Dodecadien-1-yn-3-ol, 3,7,11-trimethyl	C_15_H_24_O	20.034	73,381	1.11
27	Caryophyllene oxide	C_15_H_24_O	18.701	68,151	1.03
28	7,9-Dimethyl-8-nitrobicyclo[4.3.1]nonane		22.481	66,616	1.01
29	Isoeugenol	C_10_H_12_O_2_	14.274	65,947	1
30	Caryophyllene oxide	C_15_H_24_O	17.606	64,742	0.98
31	1-Tetracosanol	C_24_H_50_O	22.068	63,996	0.97
32	tau-Muurolol	C_15_H_26_O	19.602	60,852	0.92
33	(+)-Nerolidol	C_15_H_26_O	17.003	60,228	0.91
34	Cholesta-8,24-dien-3-ol, 4-methyl-,(3.beta,4.alpha.)-	C_28_H_46_O	17.654	51,339	0.78
35	1.bet.-Cadin-4-en-10-ol	C_15_H_26_O	18.977	51,532	0.78
36	Phenol,2-methyl-5-(1,2,2-trimethylcyclopentyl)-,(S)-	C_15_H_22_O	21.181	44,799	0.68
37	(-)-Spathulenol	C_15_H_24_O	17.453	39,071	0.59
38	Ergost-5-en-3-ol, acetate, (3.beta,24R)-	C_30_H_50_O_2_	22.769	34,906	0.53
39	alpha-Caryophyllene	C_15_H_24_	14.544	34,316	0.52
40	Cis-Z-alpha-Bisabolene epoxide	C_15_H_24_O	18.204	32,640	0.49
41	gamma-Muurolene	C_15_H_24_	15.954	29,904	0.45
42	Zingiberene	*C_15_H_24_*	15.44	20,949	0.32
43	n-Decanoic acid	C_10_H_20_O_2_	12.117	20,817	0.31
44	Bicyclo[4.1.0]-3-heptne, 2-isopropenyl-5-isopropyl-7,7-dimethyl-	C_15_H_24_	20.869	20,664	0.31
45	Copaene	C_15_H_24_	12.632	13,217	0.2
46	Copaene	C_15_H_24_	16.486	12,827	0.19
47	Cubenol	C_15_H_26_O	18.306	6420	0.1

**Table 2:** DPPH and ABTS inhibitory assay of the Crude extract and different fraction of *C.macrophylla*.

Samples Names	Concentration (µg/mL)	% DPPH Activity	IC_50_ (µg/mL)	% ABTS Activity	IC_50_ (µg/mL)
Crude	1000	92.23 ± 0.22 ns	17.72	83.13 ± 0.80 ***	19.34
	500	87.45 ± 0.90 ns		78.83 ± 0.73 ***	
	250	81.90 ± 0.60 ns		72.70 ± 0.51 ***	
	125	76.00 ± 0.30 ns		66.43 ± 0.70 ***	
	62.5	71.90 ± 0.45 ns		61.06 ± 0.70 ***	
n-Hexane	1000	87.63 ± 0.64 ***	20.76	89.37 ± 0.54 ns	16.76
	500	82.45 ± 0.5 ns		84.44 ± 0.50 ns	
	250	76.53 ± 0.4 **		77.51 ± 0.72 ***	
	125	71.42 ± 0.46 ***		72.28 ± 0.61 ***	
	62.5	65.68 ± 0.64 ***		67.46 ± 0.62 ***	
Dichloromethane	1000	93.10 ± 0.60 ns	5.34	82.33 ± 1.20 ***	4.06
	500	87.58 ± 0.63 ns		76.33 ± 0.95 ***	
	250	83.76 ± 0.71 ns		72.67 ± 0.91 ***	
	125	75.44 ± 0.58 ns		70.00 ± 0.17 ***	
	62.5	68.10 ± 0.90 *		68.60 ± 0.04 ***	
Ethyl Acetate	1000	94.40 ± 0.03 ns	7.8	86.91 ± 1.30 ***	9.54
	500	85.03 ± 2.16 ns		81.26 ± 1.27 ***	
	250	80.90 ± 1.11 ns		76.00 ± 0.30 ***	
	125	76.44 ± 0.28 ns		71.54 ± 0.50 ***	
	62.5	71.22 ± 0.47 ns		68.76 ± 0.58 ***	
Aqueous	1000	84.37 ± 0.64 ***	16.4	86.91 ± 1.30 ***	12.43
	500	80.45 ± 0.65 ***		81.26 ± 1.27 ***	
	250	73.37 ± 0.54 ***		76.00 ± 0.30 ***	
	125	67.30 ± 0.61 ***		71.54 ± 0.50 ***	
	62.5	62.42 ±0.55 ***		67.76 ± 0.58 ***	
Ascorbic Acid	1000	94.40 ± 0.03	4.32	91.90 ± 0.96	3.11
	500	85.03 ± 2.16		87.08 ± 0.47	
	250	80.90 ± 1.11		82.40 ± 0.20	
	125	76.44 ± 0.28		77.61 ± 0.43	
	62.5	71.22 ± 0.47		75.45 ± 0.90	

Data is represented as mean ± S.E.M; n = 3, * represent level of significance like; * = *p* < 0.05, ** = *p* < 0.01, *** = *p* < 0.001, ns; not significant.

**Table 3:** COX-2 and 5-LOX inhibitory assay of the crude extract and different fractions of *C. macrophylla*.

Compound Name	Concentration (µg/mL)	COX-2 Percent Inhibition	IC_50_	5-LOX Percent Inhibition	IC_50_
			(µg/mL)		(µg/mL)
Ethyl Acetate	1000	69.62 ± 0.58 ***	93.35	71.24 ± 0.79 ***	75.64
	500	63.35 ± 0.23 ***		65.43 ± 1.39 ***	
	250	57.36 ± 0.84 ***		59.48 ± 0.25 ***	
	125	52.62 ± 0.25 ***		54.47 ± 0.04 ***	
	62.5	46.16 ± 0.16 ***		47.47 ± 0.44 ***	
Crude	1000	69.67 ± 0.32 ***	130.02	67.44 ± 0.09 ***	122.79
	500	63.20 ± 0.10 ***		61.87 ± 0.39 ***	
	250	55.09 ± 0.32 ***		55.83 ± 1.07 ***	
	125	49.67 ± 1.20 ***		50.23 ± 0.44 ***	
	62.5	43.40 ± 0.25 ***		44.29 ± 0.43 ***	
n-Hexane	1000	69.58 ± 1.12 ***	249.57	71.33 ± 0.49 ***	218.83
	500	61.65 ± 1.34 ***		63.03 ± 0.23 ***	
	250	47.90 ± 0.96 ***		49.00 ± 0.58 ***	
	125	39.03 ± 0.48 ***		42.67 ± 0.89 ***	
	62.5	31.90 ± 0.48 ***		33.00 ± 1.15 ***	
Aqueous	1000	66.79 ± 0.63 ***	319.7	67.73 ± 0.03 ***	277.91
	500	59.67 ± 0.61 ***		57.42 ± 0.12 ***	
	250	41.69 ± 0.77 ***		47.39 ± 0.35 ***	
	125	35.54 ± 0.50 ***		41.36 ± 0.71 ***	
	62.5	29.00 ± 0.30 ***		29.15 ± 0.22 ***	
Dichloromethane	1000	71.02 ± 1.32 ***	72.55	77.00 ± 0.15 ***	49.52
	500	66.69 ± 0.33 ***		69.26 ± 1.55 ***	
	250	61.14 ± 0.60 ***		65.89 ± 0.49 ***	
	125	56.44 ± 0.84 ***		58.36 ± 0.71 ***	
	62.5	47.72 ± 0.48 ***		51.47 ± 0.42 ***	
Celecoxib	1000	81.85 ± 0.18	21.58		
	500	76.59 ± 0.30			
	250	69.75 ± 0.14			
	125	64.47 ± 0.49			
	62.5	61.02 ± 0.22			
Montelukast	1000			83.53 ± 0.20	17.3
	500			78.62 ± 0.17	
	250			73.42 ± 0.11	
	125			66.20 ± 0.15	
	62.5			62.00 ± 1.15	

Data symbolized as ns; non-significant, mean ± S.E.M; values are significant to the positive control; *** = *p* < 0.001 and *n* = 3.

**Table 4:** Total phenolic content and total flavonoid content *C. macrophylla* Bark.

Phytochemical Assays	*C. macrophylla* Bark
Total phenolic content (TPC) GAE/5g	18.52 ± 0.34
Total flavonoid content (TFC) QUE/5g	32.18 ± 0.52

**Table 5:** Binding energies of compounds docked with COX-2 and 5-LOX.

S. No.	Name of Compound	Binding Energies with COX-2 Enzyme	Binding Energies with 5-LOX Enzyme
1	Debezylhydrazine	−7.9	−7.5
2	n−Decanoic acid	−6.9	−5.6
3	Copaene	−4.4	−3.8
4	Aromadandrene	−5.4	−4.2
5	Isoeugenol	−6.1	−6.6
6	alpha−Caryophyllene	−5.0	−4.6
7	alpha−Curcumene	−7.0	−5.7
8	Phenol,3,5−bis(1,1−dimethylethyl)−	−7.6	−8.2
9	gamma−Muurolene	−6.0	−5.3
10	delta−Cadinene	−5.8	−5.5
11	alpha−Calacorene	−5.7	−5.1
12	Undecanoic acid	−7.3	−6.2
13	(+)−Nerolidol	−7.0	−5.0
14	(−)−Spathulenol	−4.8	−3.9
15	Caryophyllene oxide	−4.4	−4.3
16	Cholesta−8,24−dien−3−ol, 4−methyl,(3.beta.,4.alpha.)−	−6.6	−5.9
17	Epiglobulol	−5.5	−3.9
18	Cis−Z−.alpha−Bisabolene epoxide	−7.2	−5.7
19	Cubenol	−4.3	−4.6
20	Tetracyclo[6.3.2.0(2,5).0(1,8)]tridecan−9−ol, 4,4−dimethyl−	−4.3	---
21	1H−3a,7−Methanoazulene, octahydro−1,4,9,9−tetramethyl−	−4.1	---
22	alpha−Bisabolol	−6.4	−7.3
23	6,10−Dodecadien−1−yn−3−ol, 3,7,11−trimethyl	−6.3	−5.2
24	Bicyclo[4.1.0]−3−heptne, 2−isopropenyl−5−isopropyl−7,7−dimethyl−	−6.8	−5.5
25	7−Acetyl−2−hydroxy−2−methyl−5−isopropylbicyclo[4.3.0]nonane	−7.2	−8.1
26	Tetracosanol	−6.7	−6.0
27	Ergost−5−en−3−ol, acetate, (3.beta,24R)−	−6.8	−6.1
28	4,4,8−Trimethyltricyclo[6.3.1.0(1,5)]dodecane−2,9−diol	---	−5.5
29	Tridecanoic acid, methyl ester	−7.3	−7.1
30	Docosanoic acid, ethyl ester	−7.0	−6.2
31	9,12−Octadecadecenoicacid, methyl ester, (*E*,*E*)−	−6.2	−5.5
32	6−Octadecenoic acid, methyl ester, (Z)−	−8.1	−5.3
33	10−Undecenal	−6.3	−5.4
34	9−Octadecynoicacid	−6.8	−6.5
35	tauCadinol	−6.3	−6.4
36	Copaene	−4.4	−3.8
37	alpha−Cadinol	−6.8	−5.1

2−methyl−5−isopropylbicyclo [4.3.0] nonane and 6−Octadenoic acid, methyl ester are the most potent compounds according to their binding energies. Docking study of these compounds further validates in vitro anti-inflammatory results.

## Data Availability

Data will be available upon request to the corresponding author.

## Supplementary Materials

The following supporting information can be downloaded at: https://www.mdpi.com/article/10.3390/molecules27134081/s1, Figure S1: Spectral analysis of Ethyl acetate fractions of *C. macrophylla* Bark.

## Sample Availability

Samples of the extracts are available with the permission of the corresponding author.
