# Supplementary material for: Phytochemical Profiling, Anti-Inflammatory, Anti-Oxidant and In-Silico Approach of Cornus macrophylla Bioss (Bark)
Source: Molecules. 2022 Jun 24;27(13):4081. doi: 10.3390/molecules27134081 (PMC9268425; doi:10.3390/molecules27134081)
Supplement: Supplementary file 1 [file molecules-27-04081-s001.zip › molecules-1690125-Supplementary.pdf]

Article

Phytochemical Profiling, Anti-inflammatory, Anti-oxidant and In-silico Approach of *Cornus macrophylla* Bioss (Bark).

Ali khan[1], Aini Pervaiz[1], Bushra Ansari[2], Riaz Ullah[3], Syed Muhammad Mukarram Shah*[1], Haroon Khan[2], Muhammad Saeed Jan[1], Fida Hussain[1], Mohammad Ijaz Khan[1], Ghadeer M. Albadrani[4], Ahmed E. Altyar[5] and Mohamed M. Abdel-Daim[6, 7]

[1]Department of Pharmacy, University of Swabi, Swabi, KP, Pakistan
[2]Departement of Pharmacy, Abdul Wali Khan University, KP, Mardan
[3]Department of Pharmacognosy, College of Pharmacy, King Saud University, Riyadh 11451, Saudi Arabia
[4]Department of Biology, College of Science, Princess Nourah bint Abdulrahman University, B.O. Box 84428, Riyadh 11671, Saudi Arabia; gmalbadrani@pnu.edu.sa
[5]Department Pharmacy Practice, Faculty of Pharmacy, King Abdulaziz University, Jeddah 21589, Saudi Arabia.
aealtyar@kau.edu.sa
[6]Department of Pharmaceutical Sciences, Pharmacy Program, Batterjee Medical College, P.O. Box 6231, Jeddah 21442, Saudi Arabia; abdeldaim.m@vet.suez.edu.eg (M.M.A.-D.)
[7]Pharmacology Department, Faculty of Veterinary Medicine, Suez Canal University, Ismailia 41522, Egypt *

* Correspondence: Prof. Dr. Syed Muhammad Mukarram Shah, Department of Pharmacy, University of Swabi, Swabi, KP, Pakistan. Email: mukaramshah@uoswabi.edu.pk
Contact: 0092 333 9256603

Abstract: *Cornus macrophylla* (*C. macrophylla*) is from the family cornaceae. It is locally known as khadang and is used for the treatment of different diseases such as analgesic, tonic, diuretic, malaria, inflammation, allergy, infections, cancer, diabetes, and as a lipid peroxidative. The crude extract and different fractions of *C. Macrophylla* were evaluated by gas chromatography and mass spectroscopy (GC-MS), which identified the most potent bioactive phytochemicals. The antioxidant ability of *C. macrophylla* was studiedby2,2'-azino-bis-3-ethylbenzthiazoline-6-sulfonic acid (ABTS) and 1,1 diphenyl-2-picryl-hydrazyl (DPPH) methods and in vitro anti-inflammatory activity was assessed by using COX-2 (Cyclooxygenase-2) and 5-LOX (5-lipoxygenase) assays. The molecular docking was carried out by using molecular operating environment (MOE) software. The GC-MS study of *C. macrophylla* confirmed forty-eight compounds in ethylacetate (Et.AC) fraction and revealed that Et.AC fraction was the most active fraction. The antioxidant ability of the Et.AC fraction showed an IC_{50} value of 09.54 μg/ml and 7.8 μg/ml against ABTS and DPPH assay respectively. Among all the fractions of *C. macrophylla,* Et.AC showed excellent activity against COX-2 and 5-LOX. The observed IC_{50} values were 93.35 μg/ml against COX-2 and 75.64 μg/ml for 5-LOX respectively. Molecular docking studies supported these in vitro results and confirmed the anti-inflammatory potential of *C. macrophylla. C. macrophylla* has promising potential as a source for the development of new drugs against inflammation in the future.

Keywords: GC-MS; anti-inflammatory; antioxidant; *Cornus macrophylla*;

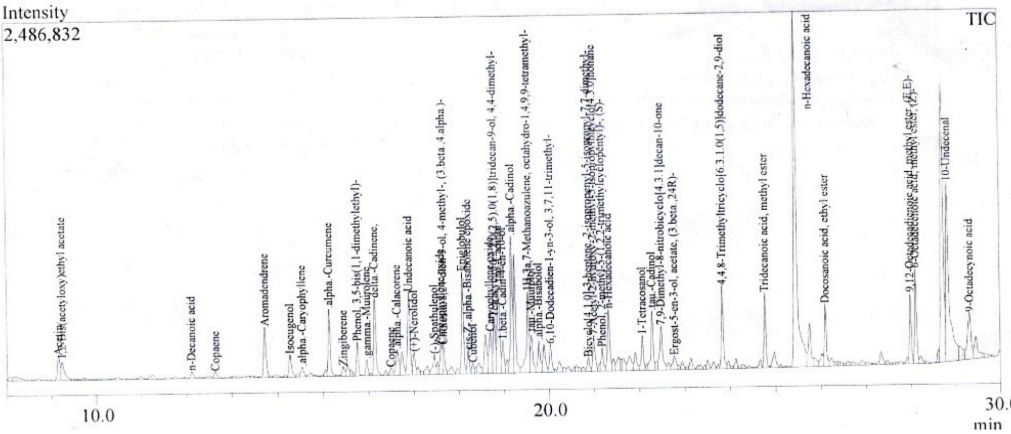

Figure S1. Spectral analysis of ethyl acetate fractions of *C. macrophylla* bark.
